# Phylogenomic Inference Suggests Differential Deep Time Phylogenetic Signals from Nuclear and Organellar Genomes in Gymnosperms

**DOI:** 10.3390/plants14091335

**Published:** 2025-04-28

**Authors:** Yu-En Lin, Chung-Shien Wu, Yu-Wei Wu, Shu-Miaw Chaw

**Affiliations:** 1 Department of Biochemical Science and Technology, National Taiwan University, Taipei 106319, Taiwan; wayne0218888@gmail.com; 2 Biodiversity Research Center, Academia Sinica, Nankang Campus, Taipei 11529, Taiwan; d92625002@gmail.com; 3 Graduate Institute of Medical Bioinformatics, College of Medical Science and Technology, Taipei Medical University, Taipei 11030, Taiwan; yuwei.wu@tmu.edu.tw

**Keywords:** RNA editing, cycads, ginkgo, conifers, gnetophytes, phylogenomics, *Nageia*, cytonuclear incongruence, mitogenome, plastome

## Abstract

The living gymnosperms include about 1200 species in five major groups: cycads, ginkgo, gnetophytes, Pinaceae (conifers I), and cupressophytes (conifers II). Molecular phylogenetic studies have yet to reach a unanimously agreed-upon relationship among them. Moreover, cytonuclear phylogenetic incongruence has been repeatedly observed in gymnosperms. We collated a comprehensive dataset from available genomes of 17 gymnosperms across the five major groups and added our own high-quality assembly of a species from Podocarpaceae (the second largest conifer family) to increase sampling width. We used these data to infer reconciled nuclear species phylogenies using two separate methods to ensure the robustness of our conclusions. We also reconstructed organelle phylogenomic trees from 42 mitochondrial and 82 plastid genes from 38 and 289 gymnosperm species across the five major groups, respectively. Our nuclear phylogeny consistently recovers the Ginkgo–cycads clade as the first lineage split from other gymnosperm clades and the Pinaceae as sister to gnetophytes (the Gnepines hypothesis). In contrast, the mitochondrial tree places cycads as the earliest lineage in gymnosperms and gnetophytes as sister to cupressophytes (the Gnecup hypothesis) while the plastomic tree supports the Ginkgo–cycads clade and gnetophytes as the sister to cupressophytes. We also examined the effect of mitochondrial RNA editing sites on the gymnosperm phylogeny by manipulating the nucleotide and amino acid sequences at these sites. Only complete removal of editing sites has an effect on phylogenetic inference, leading to a closer congruence between mitogenomic and nuclear phylogenies. This suggests that RNA editing sites carry a phylogenetic signal with distinct evolutionary traits.

## 1. Introduction

Gymnosperms are one of the two clades of extant seed plants that originated in the Middle-Devonian period about 385 million years ago [1,2]. In contrast to angiosperms, which comprise some 350,000 species, gymnosperms are much less diverse and include only 1174 species [2]. Nonetheless, gymnosperms dominate huge swaths of the Earth’s landmass, especially the conifers (Pinaceae and Cupressaceae) in the Northern Hemisphere and most Araucariaceae and Podocarpaceae genera in the Southern Hemisphere [3]. Moreover, gymnosperms exhibit diverse growth forms, predominantly as trees, such as pines (*Pinus*) and firs (*Abies*). Some species, like junipers (*Juniperus*), adopt a shrub-like form. While rare, a few gymnosperms, notably species within the genus *Gnetum*, exhibit vine-like growth forms. These varied growth forms reflect the adaptability of gymnosperms to a diverse range of ecological niches, from canopies to understories and from rainforests to deserts [4,5,6]. Consequently, gymnosperms are of great economic and ecological importance in several countries. For example, many *Cupressaceae* (cypress family), including false cypress (*Chamaecyparis*), China fir (*Cunninghamia*), bald cypress (*Taxodium*), and arborvitae (*Thuja*), are valuable as both timber sources and ornamentals. The yew family (*Taxaceae*), including about 30 species in six genera, produces taxane compounds useful in anticancer therapies [7].

**Figure 1:**
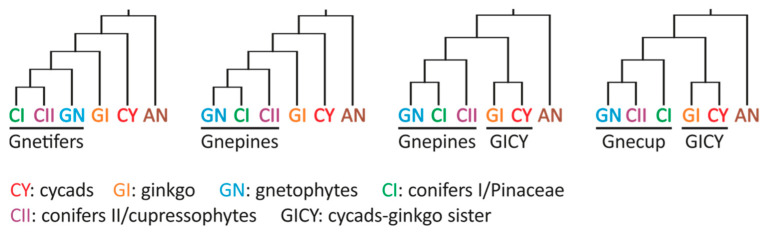
Combinations of the four major hypotheses concerning the phylogenetic relationships among the five extant gymnosperm groups. Angiosperms (AN) were the designated sister group of gymnosperms.

Advances in sequencing, alignment, and clustering methods starting in the 1990s prompted a reassessment of the classical taxonomic relationships among gymnosperms using molecular data. These analyses categorized gymnosperms into five widely accepted groups: cycads (*Cycadales*), ginkgo (*Ginkgoales*), gnetophytes (*Gnetales*), pine family (*Pinales* or conifers I), and cupressophytes (conifers II) [8,9]. The phylogenetic relationships among these groups are a subject of extensive debate [10], comparable to the controversies surrounding primitive (or early divergent) dicots, monocots, and eudicots. The gymnosperm group monophyly and the placements of ginkgo and gnetophytes were once highly contentious. The initial molecular phylogenetics studies proposed five alternative hypotheses: (1) The extant gymnosperms comprise a monophyletic group; (2) Cycads and ginkgo are sister groups (i.e., the Ginkgo–cycads hypothesis); (3) *Gnetophytes* are sister to all conifers (Gnetifer hypothesis); (4) *Gnetophytes* are sister to *Pinaceae* (the Gnepines hypothesis); and (5) Gnetophytes are sister to cupressophytes (the Gnecup hypothesis) (Figure 1) [8,9,11].

Substantial progress in understanding angiosperm evolution is driven by extensive nuclear phylogenomics studies [12,13]. Although a few recent studies have analyzed the gymnosperm phylogeny using large transcriptomic datasets [14,15], this line of investigation remains relatively underdeveloped. Nuclear genome sequencing is challenging in gymnosperms due to their large genomes (ca. 5–28 Gb [16]), with exceptionally long genes, introns, and many repeated sequences [17,18,19,20,21]. Fortunately, the advancements in sequencing technology over the past five years have made whole-genome assemblies more feasible and cost-effective.

Phylogenomic trees based on whole nuclear genomes are largely inferred from single-copy orthologous genes, excluding the numerous genes including multiple paralogs and thus only accounting for a small, and possibly biased, portion of the phylogenetic signal accumulated in genomes during evolution. Moreover, some phylogenomic trees are constructed from transcriptomes [14,15]. This can lead to several problems: (1) some transcripts are not in full length, causing sampling bias or alignment errors; (2) transcripts at low levels might lead to inaccurate assembly (or come from DNA contamination [22]); and (3) transcripts represent only a partial set of genes specific to cell types, developmental stages, and growing conditions sampled. To address these issues, we gathered 20 well-assembled seed plant nuclear genomes and incorporated two phylogeny inference strategies, ASTRAL-Pro2 (ASTRAL for Paralogs and Orthologs) and SpeciesRax [23,24,25], into this study. These recently developed gene tree-based methods can account for gene duplications, losses, transfers, and, to some degree, incomplete lineage sorting by reconciling multiple-copy gene families when building species trees.

In addition to nuclear genomes, organelle markers/genomes have been frequently used to resolve plant phylogenetic relationships at different taxonomic levels [8,9,26]. As the amount of nuclear and organellar data increases, the number of cases of discordance in phylogenies between nuclear and organellar trees, known as cytonuclear incongruence, has likewise grown (see review in Kao et al., 2022) [27]. For instance, plastomes were widely used in green plant phylogenetics for their small size, predominantly uniparental inheritance (mostly paternal in gymnosperms), stable genomic structure, resistance to recombination, and generally low nucleotide substitution rates [28,29,30,31,32,33,34,35,36,37]. However, in gymnosperms, plastid–nuclear incongruence has been consistently observed since the earliest study [38]. These discrepancies contributed, among other things, to the continuing controversy over the placement of ginkgo and gnetophytes [9,39,40,41,42,43,44,45,46]. To date, studies on gymnosperm plastome phylogeny have unanimously agreed on the Ginkgo–cycads sisterhood. Nonetheless, the position of gnetophytes is still debated, with earlier studies supporting either the Gnepines or Gnecup hypothesis (see review by Chaw et al., 2018) [30].

In contrast to the extensive studies on gymnosperm phylogeny using plastomes, mitogenomic phylogeny has received significantly less attention. This can be best attributed to the highly variable size, complex structure, genome rearrangements, and intergenomic interactions in plant mitogenomes [47,48,49,50,51,52,53]. These complicated genomic features render the assembly and syntenic analysis of mitogenomes difficult. As a result, most previous studies only extracted and combined the 40/41 conserved genes in plant mitogenomes for phylogenetic inference (e.g., Liu et al., 2014) [54]. However, since plant mitogenomes have a slow substitution rate, conserved gene numbers, and rearrangement in some gene groups, for example, *nad5-nad4-nad2* [55,56,57], it was suggested that plant mitogenomes can harbor ancient phylogenetic signals in bryophytes and other basal land plants [55]. Therefore, in this study, we consider using mitogenomes to help inform gymnosperm phylogeny reconstruction.

Another remarkable feature of gymnosperm mitogenomes is the prevalence of RNA editing sites [58]. RNA editing in plant organelles can correct mutated codons in a transcript to restore conserved amino acids [59,60,61,62]. Since mitogenomes evolve slowly, RNA editing sites may contribute to sequence variation, impacting mitophylogeny estimation and potentially causing cytonuclear incongruence. Consequently, many prior studies have tried to evaluate the impact of RNA editing sites on mitogenome phylogenetic inference. Hiesel et al. (1994) favored the use of cDNA rather than DNA to infer phylogeny, as it takes into account the corrected amino acid sequences [63]. Later, Bowe and dePamphilis (1996) found that RNA editing sites did not affect phylogenetic inference and suggested the use of either (but not both) DNA or cDNA for tree building [64]. However, subsequent comparative phylogenetics analyses argued that RNA editing sites carry important phylogenetic signals and advocated for the use of genomic DNA in mitophylogenomics [65,66,67]. Nevertheless, some studies restore the conserved amino acids or mitigate the effects of RNA editing by changing RNA editing sites from C back to T or even completely removing them [68,69,70,71,72,73,74,75].

In this study, we surveyed reference-level nuclear genomes of 18 gymnosperm species, including 17 published previously (Table 1; as of 31 July 2024), and reported the first high-quality draft genome from *Nageia nagi* in the podocarp family (*Podocarpaceae*, details in Supplementary Information). *Podocarpaceae*, with 19 genera and 187 species, mainly found in the Southern Hemisphere, is the largest and most diverse group in the conifers II clade [76]. Incorporation of the first podocarp genome should improve taxonomic sampling of cupressophytes and fill in the gaps in our understanding of nuclear genomic variation in gymnosperms. We carried out two gene tree-based phylogenetic inference strategies to reconstruct and compare two nuclear phylogenetic trees, and assess the influence of these inference methods on cytonuclear incongruence. Furthermore, we evaluated the impact of RNA editing on mito-nuclear incongruence and consolidated our previous findings regarding the plastid–nuclear incongruent placements of gnetophytes to shed light on the underlying causes of phylogenetic discordance across the five gymnosperm clades.

## 2. Results

We used nuclear, mitochondrial, and plastid genomic datasets to separately infer species trees. To explore the effect of mitochondrial RNA editing sites on phylogenetic inference, we also gathered a smaller dataset that includes 13 gymnosperm mitogenomes (ME datasets) where C-to-U editing sites in protein-coding genes were verified using transcriptomics (Table S4). Two basal angiosperms, *Amborella* and *Nymphaea*, were used as the outgroups when inferring phylogenetic trees from each dataset.

### 2.1. Multiple-Copy Nuclear Gene Families Support the Ginkgo–Cycads Sister Relationship and the Gnepines Hypothesis

**Figure 2:**
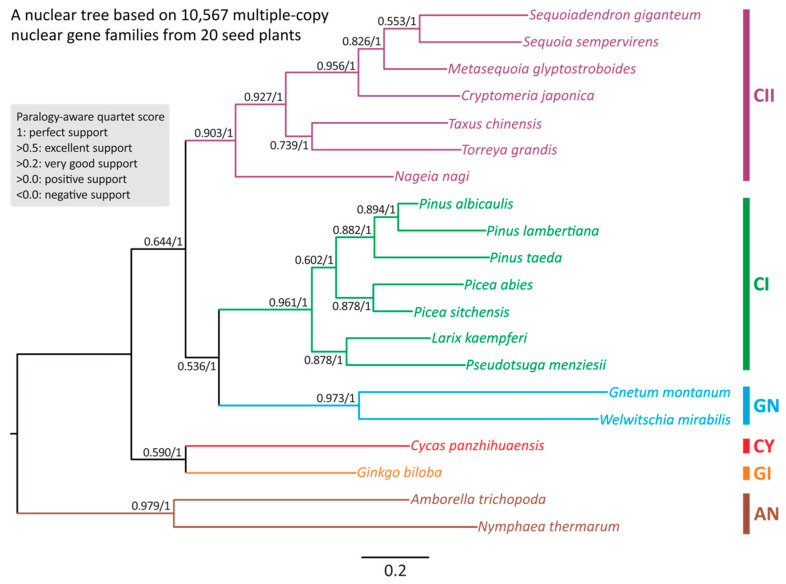
Species trees inferred from 10,567 multiple-copy nuclear gene families across 18 gymnosperms and two angiosperms. The tree framework shown here was constructed using SpeciesRax. Values along branches denote EQPIC scores (before the slashes) and bootstrap support (after the slashes) estimated using SpeciesRax and ASTRAL-Pro2, respectively. AN: angiosperms; GI: ginkgo; CY: cycads; GN: gnetophytes; CI: conifers I; CII: conifers II.

We were unable to use single-copy orthologs to reconstruct nuclear phylogenetic trees because their number drops drastically with increasing taxon count (Figure S4). We therefore used 10,567 (out of more than 40,000) multi-copy gene families that are common to the 18 sampled gymnosperm species (Table 1). We used SpeciesRax and ASTRAL-Pro2 methods to reconstruct the species phylogeny, obtaining identical results with both (Figure 2).

As expected, *Amborella* and *Nymphaea* together form an outgroup clade. At the level of taxonomical orders, our nuclear tree includes two sister clades: the so-called “Ginkgo–cycads sister” and “Gnepines” hypotheses in the gymnosperm phylogeny (Figure 2). SpeciesRax estimates robust extended quadripartition internode certainty (EQP-IC) scores at all nodes, suggesting that the quartets of gene trees also support the two sister clades within the species tree. The strongly supported nodes lead to (1) the Ginkgo–cycads clade (0.59); (2) the three sub-clades: gnetophytes (EQP-IC = 0.973), conifers I (0.961), and conifers II (0.903); and (3) the gnetophytes–conifers I (the Gnepines subclade) (0.536) clade. Notably, clades (1) and (3) also received 100% bootstrap support in ASTRAL-Pro2 (Figure 2). We thus infer that during gymnosperm evolution (1) the Ginkgo–cycads clade was the first to diverge from the other gymnosperm groups and that (2) *Pinaceae* (or conifers I clade) is sister to the gnetophytes rather than the conifers II clade. Therefore, the group commonly known as conifers is not monophyletic.

### 2.2. Discordant Mitochondrial and Plastid Phylogenomic Trees

The ML tree inferred from both the Mito and Plastid datasets recovered cycads, gnetophytes, conifers I, and conifers II each as a monophyletic clade with strong support (all BS > 99%; Figures S5 and S6). In addition, the mitochondrial tree topology suggests that (1) cycads diverged first, followed by ginkgo, and that (2) the “Gnepines” topology is well resolved (BS = 90%, Figure S5). In stark contrast, our plastid tree resolves the “Gnecup” topology and the “Ginkgo–cycads” clade with 100% and 98% bootstrap values, respectively (Figure S6). Our results demonstrate that gymnosperm mitochondrial and plastid phylogenies are not only discordant with each other but also differ from the nuclear phylogenomic tree.

### 2.3. Mitochondrial RNA Editing Sites Influence on Tree Topology

**Figure 3:**
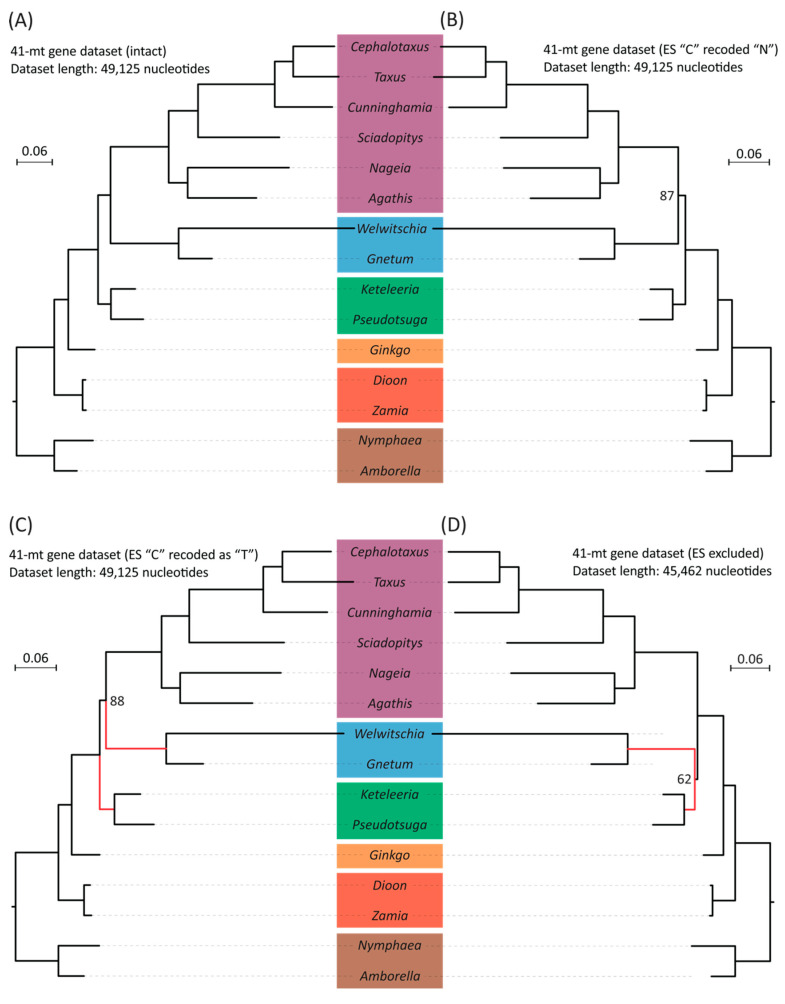
Comparisons of species trees based on four datasets generated from concatenated nucleotide alignments of 41 mt protein-coding genes with and without modifications: (**A**) A maximum likelihood (ML) tree inferred from the dataset without modification. (**B**) An ML tree inferred from the dataset where editing sites were replaced with “N” (=missing data). (**C**) An ML tree inferred from the dataset where edited C nucleotides were recoded as “T” (as in the RNA transcripts). (**D**) An ML tree was inferred from the dataset where alignment positions were removed if they contained an edited nucleotide in any taxon. Bootstrap values are indicated if they are smaller than 100%. The branch length scale bar represents 0.06 substitutions per site. Red lines highlight the placement changes of gnetophytes (here represented by *Welwitschia* and *Gnetum*) between (**C**) and (**D**) trees.

To test if RNA editing sites affect the phylogenetic tree topology, we inferred mitogenomic trees with or without these sites. We needed mitochondrial transcriptome data to find these editing sites. Therefore, the ME datasets only include 13 gymnosperm species where such data are available. Despite the reduction in taxon count, this set still includes representative species from each of the five extant gymnosperm groups. The RNA editing site numbers vary among genomes, ranging from 99 (*Welwitschia*) to 1299 (*Keteleeria*) (Figure S7). RNA editing changes some C nucleotides to U in seed plants. It functions as a repair system for maintaining normal mitochondrial protein function, masking some genomic mutations. The sites involved are thus likely under relaxed constraint and might provide a misleading phylogenetic signal. Replacing these editing sites with missing data (“N”) or thymine bases (“T”) that reflect their translated sequence would then change the phylogenetic tree topology. However, we do not observe that trees inferred from ME datasets with C-to-N and C-to-T replacements yield identical topologies, both supporting the “Gnecup” clade, and are the same as the full unaltered dataset phylogeny (Figure 3A–C). However, excluding all RNA editing sites resolved the “Gnepines” clade with decreased support (BS = 62%) (Figure 3D). Nevertheless, the placement of cycads remains unchanged.

**Figure 4:**
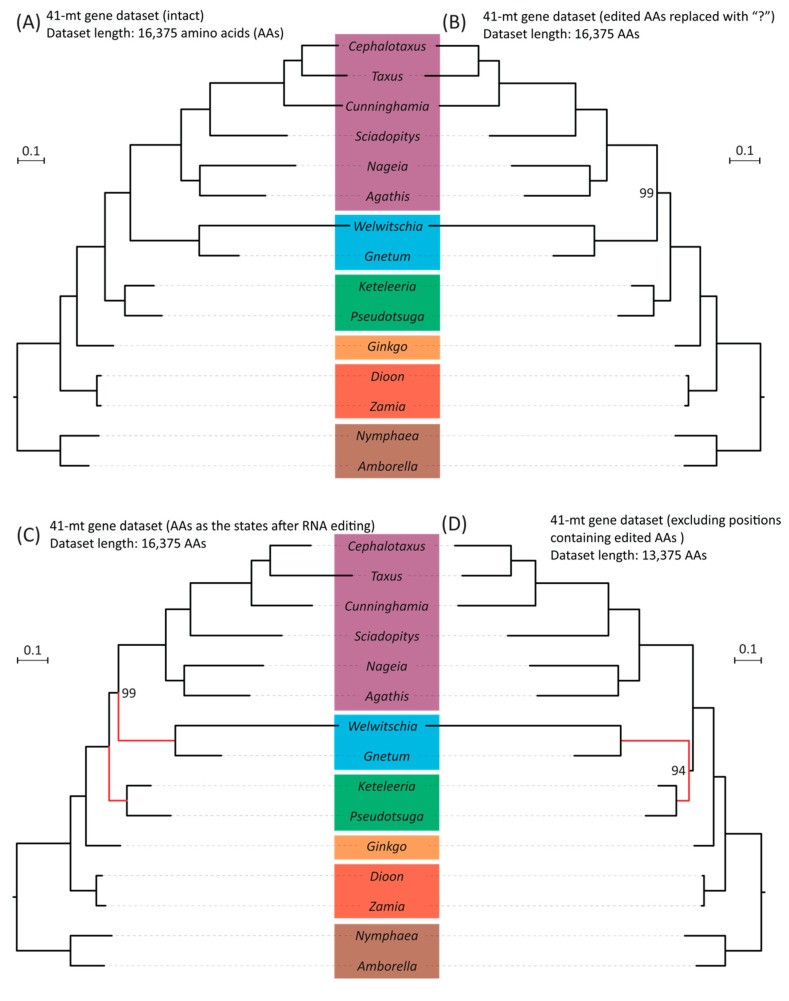
Comparisons of species trees based on four datasets generated from a concatenated amino acid alignment of 41 mt genes with and without modifications: (**A**) A ML tree inferred from the dataset without modification. (**B**) An ML tree inferred from the dataset where amino acids affected by RNA editing (including both synonymous and non-synonymous editing) were replaced with “?”. (**C**) An ML tree inferred from the dataset with amino acids recoded according to the state after RNA editing. (**D**) An ML tree inferred from the dataset with the aligned positions containing amino acids affected by editing at either synonymous or non-synonymous sites completely removed. Bootstrap values are indicated if they are smaller than 100%. The branch length scale bar represents 0.1 substitutions per site. Red lines highlight the placement changes of gnetophytes (here represented by *Welwitschia* and *Gnetum*) between (**C**) and (**D**) trees.

Because most RNA editing leads to changes in protein sequences, we re-inferred phylogenetic trees using amino acid sequences. We modified the original dataset, in line with the manipulations of the DNA sequences, as follows: (1) amino acids were replaced with “?” if their codons harbored editing sites; (2) all editing sites were recoded as “T” before translation, generating amino acids sequences that are produced in vivo; and (3) positions in the alignments were completely excluded if they contained amino acids affected by either synonymous or nonsynonymous editing in any sampled taxon. Phylogenies estimated from the original and the first two modified datasets are the same (Figure 4A–C). The only difference is that the inference based on the dataset without amino acids affected by RNA editing isolates “Gnepines” into a monophyletic clade with strong support (BS = 94%, Figure 4D). In contrast, the placement of ginkgo is insensitive to the modifications of sampled taxa numbers and RNA editing sites. All mitochondrial trees, inferred from either nucleotide or amino acid sequences, strongly indicate that ginkgo diverged after cycads and is sister to the clade comprising gnetophytes, conifers I, and conifers II (Figure S5, Figure 3 and Figure 4; with all BS = 100%).

In summary, we analyzed three comprehensive datasets, a nuclear and two organelle genomes, of gymnosperms separately to examine the causes of cytonuclear incongruence. Using two separate methods, SpeciesRax and ASTRAL-Pro2, we obtained congruent gymnosperm nuclear phylogenomic trees. ML trees from gymnosperm mitogenomes and plastomes recovered some of the same clades but were not completely in agreement with the nuclear genome-based phylogeny. With ME datasets, we also saw that eliminating RNA editing sites from consideration restored “Gen–pines” monophyly. Keeping these sites, regardless of the treatment of their nucleotide or amino acid states, makes this clade paraphyletic.

## 3. Discussion

Plant nuclear genomes generally mutate at higher rates than the two organelle (or cytoplasmic) genomes [56]. Additionally, while nuclear genomes undergo sexual reproduction and recombination, organelle genomes are generally uniparentally inherited without sexual recombination and thus less genetically variable. The absence of recombination can lead to a build-up of harmful mutations and eventually the meltdown of cytoplasmic genomes, a phenomenon known as Muller’s ratchet [77,78,79], but this process can be slowed down by the low mutation rate in organellar genomes [56].

Interestingly, some plant species (e.g., *Pelargonium*, *Plantago*, and *Silene*) exhibit extraordinarily accelerated mutation rates in their organellar genomes. In these taxa, biparental inheritance of plastids can occur under mild environmental stress, challenging the long-held belief that organelles are strictly asexual [80]. These discoveries imply that cyto-nuclear phylogenomic incongruence might be common in plants, as nuclear and organellar genomes follow fundamentally different inheritance patterns. In addition, nuclear genes regulate the function and division of organellar genomes.

Earlier studies attributed the cause of cytonuclear incongruence to a variety of processes including long-branch attraction (LBA), incomplete lineage sorting, introgression, gene duplication and loss, distinct organelle inheritance modes, or analytical factors such as sample size and taxon sampling strategies [27,67,81,82,83,84,85,86,87,88,89,90,91,92,93,94]. In this study, we further scrutinize the causes of cytonuclear incongruence in gymnosperms. To do so, we first constructed a nuclear phylogenomic tree with minimized noise from incomplete lineage sorting, gene duplication and loss, and insufficient sample size. Our objective was to reduce systematic errors in the nuclear phylogenetic inference so that we can justify whether the observed incongruence originates from intrinsic evolutionary processes of organellar genomes or from methodological artifacts.

Using the first comprehensive nuclear genome dataset to include *Nageia* (Podocarpaceae), we applied two gene tree-based phylogenetic inference methods: ASTRAL-Pro2 and SpeciesRax. Traditional concatenation methods combine per-gene alignments into a single supermatrix to infer a species tree. Since the concatenation method only works well with accurate orthology inference [95], it often fails when the evolution of genes deviates from that of species due to incomplete lineage sorting or events like duplications, loss, and transfers [96]. On the other hand, gene family tree methods, like ASTRAL-Pro2 and SpeciesRax, preserve the distinct evolutionary history within individual gene families and thus can explicitly account for the underlying processes causing discordance. Specifically, ASTRAL-Pro2 operates under the multispecies coalescent framework, which uses the statistical distribution of gene trees (summarized as quartets) to reduce the influence of incomplete lineage sorting. This method is particularly powerful when multiple-copy nuclear gene families are involved, as it leverages the coalescent process to “average out” the discordance introduced by incomplete lineage sorting. In contrast, SpeciesRax employs a maximum likelihood approach that explicitly models gene duplication, loss, and horizontal transfer events. By incorporating information from both orthologs and paralogs, SpeciesRax is designed to address the complexities of gene family evolution. However, it does not explicitly mitigate the effects of incomplete lineage sorting as the coalescent-based methods do. Together, these two approaches provide a robust alternative to concatenation-based methods by directly modeling the heterogeneous evolutionary processes that shape gene trees [23,24,25,97].

Using both methods, we obtained identical topologies of the rooted trees on nuclear data. Our results agree with previous studies that include inferences of rooted as well as unrooted trees [9,14,15]. Therefore, nuclear phylogenomics of living gymnosperms has reached a consensus that places ginkgo and cycads as the earliest-diverging lineage, followed by two sister groups: Gnepines and cupressophytes (Figure 2). Furthermore, mounting molecular phylogenetic evidence over the past two decades also supports Pinaceae and gnetophyte monophyly. We thus propose replacing the term “conifers” with Conifers I (Pinaceae) and Conifers II (cupressophytes) in future research for clarity, since conifers are consistently paraphyletic.

Our analysis of the gymnosperm plastome phylogeny supports the Ginkgo–cycads topology and the Gnecup topology, in contrast to the nuclear-derived Gnepines topology (Table 2, Figure 2 and Figure S6). Numerous factors have been proposed to interpret such incongruence, including incomplete lineage sorting [98], long-branch attraction (LBA) [41,43,99,100], and chloroplast capture [27,101,102,103,104,105], to name a few. We previously reported significantly accelerated nucleotide substitution rates in gnetophytes, potentially leading to long-branch attraction artifacts in phylogenetic reconstruction [41,43]. Multiple methods that alleviate this artifact restore the Gnepines topology in plastome trees, suggesting that long-branch attraction plays at least a role in mis-inference [41,42,43,106]. However, additional analyses would be needed to eliminate or confirm other potential sources of phylogenetic inconsistencies between analyses based on nuclear and plastid data.

Phylogenies reconstructed from mitochondrial datasets differ from both nuclear- and plastid-based trees. In particular, the mitogenomic phylogeny includes cycads as sister to the remaining gymnosperms (Table 2, Figure 2, Figures S5 and S6). Given that RNA editing sites have been proposed as a potential source of phylogenomic incongruence, we further investigated whether modifications of the RNA editing sites at either the genomic or protein level could address the observed mito-nuclear discrepancies. Nevertheless, using transcriptomic-verified ME datasets, our mitogenome species trees show no topological difference before and after the substitution of RNA editing sites (Figure 4A–C), which indicates that the substitution alone is not enough to eliminate the effect of RNA editing, as suggested earlier by other authors [74,75]. This observation is consistent with the notion that the abundance of RNA editing sites correlates with the irregular substitution rates and pronounced nucleotide compositional biases, such as an overrepresentation of pyrimidines, even after substitution [58,62,67,74,75]. In addition, RNA editing frequently produces homoplastic changes, as similar edited states may arise independently in different lineages, thereby introducing convergent noise that confounds the true phylogenetic signal [64]. Moreover, different taxa may exhibit varying efficiencies or patterns of RNA editing. As a result, a uniform substitution does not capture the full extent of the underlying evolutionary variability (Figure S7) [60]. Consequently, only the complete removal of RNA editing sites can mitigate the influence and restore the Gnepines relationship that resembles the nuclear phylogenomic tree topology at both the genomic and protein level (Figure 3D and Figure 4D). Furthermore, there is the possibility that the slow substitution rate of mitogenomes allows them to carry deep historical signals from ancient events, such as hybridization [70,71,85]. This might explain why excluding these sites did not alter the sister relationship of cycads to the remaining gymnosperms (Figure 3D and Figure 4D) [75,107].

In summary, we suggest that the intrinsic properties of RNA editing sites, like their rapid, biased evolution and convergent behavior, contribute to the observed mito-nuclear incongruence, and that the complete exclusion of them is necessary to mitigate misleading effects. Nonetheless, RNA editing sites in gymnosperms are too few to bias the inference from unedited sites that preserve mitogenomes’ deep and strong phylogenetic signals from ancient hybridization events. We thus see a general preservation of tree topology even after eliminating editing sites, other than the restoration in the placement of gnetophytes [69]. We also note that the “Gnepines” relationship recovered from the full mitochondrial dataset (Figure S5) was not resolved in the ME datasets that contain only 13 gymnosperm species (Figure 4A). This suggests that a reduction in taxon sampling can also affect mitochondrial tree topology.

## 4. Materials and Methods

### 4.1. Data Access

Nuclear genomes and their annotations were downloaded from the National Center for Biotechnology Information (NCBI, https://www.ncbi.nlm.nih.gov/ (accessed on 31 July 2024)), the China National Center for Bioinformation (CNCB, https://ngdc.cncb.ac.cn), and the TreeGenes (https://treegenesdb.org/) databases. Incorporating our newly sequenced *Nageia* draft genome (see Supplementary Information, Figures S1–S4, Tables S1 and S2 [108,109,110,111,112,113,114,115,116,117]), we gathered 18 gymnosperm and two angiosperm genomes that were well-annotated and assembled to scaffold/chromosomal levels (Nu dataset; Table 1). We used AGAT 1.4.0 (https://github.com/NBISweden/AGAT (accessed on 31 July 2024)) to retrieve amino acid sequences of annotated genes from these 20 nuclear genomes. We also gathered amino acid sequences from publicly available mitochondrial and plastid genomes/scaffolds, comprising the Mito and Plastid datasets with the former representing 38 (Table S3) and the latter 289 (Table S4) gymnosperm species. Both datasets also include two angiosperms as the outgroups.

### 4.2. Classification of Nuclear Multiple-Copy Gene Families and Construction of Gene and Species Trees

OrthoFinder v3.0.1b1 was used to identify orthologous genes in the 20 sampled nuclear genomes [118]. Only one single-copy and 43,333 multiple-copy nuclear gene families were obtained. To save on computing time, we discarded gene families that are shared by fewer than 10 taxa (i.e., fewer than half of the 20 sampled taxa). This resulted in 10,567 gene families retained for downstream analyses. After using MUSCLE 3.8.1551 to align the sequences [119], the resulting alignments were used to infer gene trees with ParGenes v1.1.2 and its “-m” option for automatically determining the best-fit model [120]. The generated gene trees were pooled before reconciling into species trees using Astral-Pro 2 v1.15.2.4 or SpeciesRax v2.1.3 [23,24,25].

### 4.3. Construction of Mitochondrial and Plastid Trees

The amino acid sequences of mitochondrial and plastid genes were aligned and concatenated into supermetrics using Geneious Prime (www.geneious.com). To better handle sequence heterogeneity in the supermetrics, each gene was designated as a partition to determine the best-fit model. We employed IQtree 2.3.6 for constructing mitochondrial and plastid trees with the “MFP+MERGE” option and 1000 nonparametric bootstrap replicates [121].

## 5. Conclusions

We present the most comprehensive dataset and analysis of gymnosperm cytonuclear incongruence to date. Our results encompass nuclear phylogenomic analyses incorporating, for the first time, a draft Podocarpaceae (from the Southern Hemisphere Conifers II clade) genome. We also construct mitogenome and plastome trees to investigate the factors underlying the observed nuclear–organellar discrepancies. Our extensive analyses suggest that gymnosperm cytonuclear incongruence is likely due to several factors: (1) distinct organelle genome inheritance modes that increase the influence from ancient phylogenetic signals; (2) insufficient taxon sampling; and (3) inference bias that stems from genomic processes producing misleading evolutionary signals, like RNA editing sites, incomplete lineage sorting, and long-branch attraction. Future studies should carefully account for these factors when interpreting discrepancies among inferences based on disparate data sources.

## Figures and Tables

**Table 1:** The available and well-annotated gymnosperm nuclear genomes (last accessed: July 2024).

Taxonomic Group	Species	BioProject Accession Number	Assembly Level	Size (Gb)
Conifers I	*Larix kaempferi*	PRJNA587041 ^N^	Scaffold	10.9
	*Picea abies*	PRJEB1822 ^N^	Scaffold	12
	*Picea sitchensis*	PRJNA304257 ^N^	Scaffold	20.5
	*Pinus albicaulis*	PRJNA1034085 ^N^	Scaffold	27.6
	*Pinus lambertiana*	PRJNA174450 ^N^	Scaffold	27.6
	*Pinus taeda*	PRJNA174450 ^N^	Scaffold	20.5
	*Pseudotsuga menziesii*	PRJNA174450 ^N^	Scaffold	14.7
Conifers II	*Cryptomeria japonica*	PRJDB13806 ^N^	Chromosome	9
	*Metasequoia glyptostroboides*	PRJCA016596 ^C^	Chromosome	8.1
	Nageia nagi *	PRJNA1179671 ^N^	Chromosome	4.3
	*Sequoia sempervirens*	PRJNA542879 ^N^	Scaffold	26.5
	*Sequoiadendron giganteum*	PRJNA541481 ^N^	Chromosome	8.1
	*Taxus chinensis*	PRJNA730337 ^N^	Chromosome	10.2
	*Torreya grandis*	PRJNA938254 ^N^	Scaffold	19.1
Cycads	*Cycas panzhihuaensis*	PRJNA734434 ^N^	Chromosome	10.5
Ginkgo	*Ginkgo biloba*	PRJCA001755 ^C^	Chromosome	9.8
Gnetophytes	*Gnetum montanum*	PRJNA339497 ^N^	Scaffold	2.1
	*Welwitschia mirabilis*	PRJCA004995 ^C^	Chromosome	6.8

* First time sequenced and reported in this project. ^N^: NCBI, ^C^: CNCB.

**Table 2:** Comparisons of phylogenomic trees inferred from different datasets on controversial clades among the five gymnosperm groups.

Genome Dataset	Ginkgo–Cycads	Gnepines	Gnecup
**Nuclear**	Yes	Yes	No
**Mito**	No	Yes	No
**Plastid**	Yes	No	Yes
**ME**-genomic	No	No	Yes
**ME**-protein	No	No	Yes
**ME**-ES * corrected	No	No	Yes
**ME**-ES * excluded	No	Yes	No

* ES: RNA editing sites.

## Data Availability

All of the raw sequence reads used in this study have been deposited in NCBI BioProject under the accession PRJNA1179671.

## Supplementary Materials

The following supporting information can be downloaded at: https://www.mdpi.com/article/10.3390/plants14091335/s1, Figure S1: Flow cytometry of DNA content in *N. nagi*; Figure S2: Accumulations of scaffolds from the longest to the shortest; Figure S3: Dot-plot analysis of the 13 longest scaffolds; Figure S4: Variation in the number of identified single-copy gene families with different strategies of taxon sampling; Figure S5: A maximum likelihood (ML) tree inferred from concatenated amino acid alignments of 42 mitochondrial genes across 38 gymnosperms; Figure S6: A maximum likelihood (ML) tree inferred from concatenated amino acid alignments of 82 plastid genes across 289 gymnosperms; Figure S7: Variation in the total number of RNA editing sites across the 13 sampled gymnosperm mitogenomes; Table S1: Assembly statistics before and after HiRise scaffolding; Table S2: BUSCO statistics; Table S3: Gymnosperm plastomes used in this study; Table S4. Gymnosperm mitogenomes/scaffolds used in this study.

## Abbreviations

NCBI	National Center for Biotechnology Information
CNCB	China National Center for Bioinformation
